# Structural Characterization of Acidic M17 Leucine Aminopeptidases from the TriTryps and Evaluation of Their Role in Nutrient Starvation in *Trypanosoma brucei*

**DOI:** 10.1128/mSphere.00226-17

**Published:** 2017-08-16

**Authors:** Jennifer Timm, Maria Valente, Daniel García-Caballero, Keith S. Wilson, Dolores González-Pacanowska

**Affiliations:** a Structural Biology Laboratory, Department of Chemistry, University of York, York, United Kingdom; b Instituto de Parasitología y Biomedicina López-Neyra, Consejo Superior de Investigaciones Científicas, Parque Tecnológico de Ciencias de la Salud, Armilla, Granada, Spain; University of Georgia

**Keywords:** *Leishmania major*, *Trypanosoma brucei*, *Trypanosoma cruzi*, crystallography, knockout, leucine aminopeptidase

## Abstract

Leucine aminopeptidase (LAP) is found in all kingdoms of life and catalyzes the metal-dependent hydrolysis of the N-terminal amino acid residue of peptide or amino acyl substrates. LAPs have been shown to participate in the N-terminal processing of certain proteins in mammalian cells and in homologous recombination and transcription regulation in bacteria, while in parasites, they are involved in host cell invasion and provision of essential amino acids for growth. The enzyme is essential for survival in *Plasmodium falciparum*, where its drug target potential has been suggested. We report here the X-ray structures of three kinetoplastid acidic LAPs (LAP-As from *Trypanosoma brucei*, *Trypanosoma cruzi*, and *Leishmania major*) which were solved in the metal-free and unliganded forms, as well as in a number of ligand complexes, providing insight into ligand binding, metal ion requirements, and oligomeric state. In addition, we analyzed mutant cells defective in LAP-A in *Trypanosoma brucei*, strongly suggesting that the enzyme is not required for the growth of this parasite either *in vitro* or *in vivo*. In procyclic cells, LAP-A was equally distributed throughout the cytoplasm, yet upon starvation, it relocalizes in particles that concentrate in the perinuclear region. Overexpression of the enzyme conferred a growth advantage when parasites were grown in leucine-deficient medium. Overall, the results suggest that in *T. brucei*, LAP-A may participate in protein degradation associated with nutrient depletion.

**IMPORTANCE** Leucine aminopeptidases (LAPs) catalyze the hydrolysis of the N-terminal amino acid of peptides and are considered potential drug targets. They are involved in multiple functions ranging from host cell invasion and provision of essential amino acids to site-specific homologous recombination and transcription regulation. In kinetoplastid parasites, there are at least three distinct LAPs. The availability of the crystal structures provides important information for drug design. Here we report the structure of the acidic LAPs from three kinetoplastids in complex with different inhibitors and explore their role in *Trypanosoma brucei* survival under various nutrient conditions. Importantly, the acidic LAP is dispensable for growth both *in vitro* and *in vivo*, an observation that questions its use as a specific drug target. While LAP-A is not essential, leucine depletion and subcellular localization studies performed under starvation conditions suggest a possible function of LAP-A in the response to nutrient restriction.

## Abstract (precis)

Leucine aminopeptidases (LAPs) catalyze the hydrolysis of the N-terminal amino acid of peptides and are considered potential drug targets. They are involved in multiple functions ranging from host cell invasion and provision of essential amino acids to site-specific homologous recombination and transcription regulation. In kinetoplastid parasites, there are at least three distinct LAPs. The availability of the crystal structures provides important information for drug design. Here we report the structure of the acidic LAPs from three kinetoplastids in complex with different inhibitors and explore their role in *Trypanosoma brucei* survival under various nutrient conditions. Importantly, the acidic LAP is dispensable for growth both *in vitro* and *in vivo*, an observation that questions its use as a specific drug target. While LAP-A is not essential, leucine depletion and subcellular localization studies performed under starvation conditions suggest a possible function of LAP-A in the response to nutrient restriction.

## INTRODUCTION

Neglected tropical diseases (NTDs) are a medically diverse group of infectious diseases mainly affecting people in the developing world. Among the NTDs are the diseases caused by the TriTryps, unicellular parasites of the order kinetoplastida, namely, *Trypanosoma brucei*, the causative agent of African sleeping sickness, *Trypanosoma cruzi*, which is responsible for Chagas’ disease, and *Leishmania* species, which cause leishmaniases. Taken together, they are responsible for the suffering of millions of people.

African sleeping sickness (or human African trypanosomiasis [HAT]), is caused by *T. brucei* parasites and is ultimately fatal if left untreated. Estimates by the World Health Organization (WHO) state that approximately 20,000 current infections with 2,804 cases were recorded in 2015 (1). Additionally, *T. brucei* and the closely related species *Trypanosoma vivax* and *Trypanosoma congolense* have huge impacts on agriculture, causing a disease called nagana in cattle and other domestic and wild animals. Chagas’ disease, caused by *T. cruzi* parasites, infects an estimated 8 million people in Central and South America. The leishmaniases are caused by parasites of the genus *Leishmania* and include different forms of the disease, i.e., cutaneous, mucocutaneous, and visceral leishmaniases. The WHO estimates 700,000 to 1 million new cases and 20,000 to 30,000 deaths annually (2).

Control of these diseases is difficult, as the parasites have complex life cycles involving insect vectors. Current treatments for HAT (3), Chagas’ disease (4), and leishmaniasis (5) are highly toxic, the treatment regimens are difficult to implement, and the availability of some of them is limited. Emerging resistance and limited efficacy of available drugs emphasize the need for the development of new drugs with novel targets.

Peptidases are central enzymes in parasite metabolism, and some were shown to be potential drug targets in parasitic protozoa (6–8), as in the case of *Plasmodium falciparum*, the causative agent of malaria. Furthermore, the aminopeptidase inhibitor arphamenin A was shown to inhibit the growth of *T. brucei* subsp. *brucei* cells *in vitro*, suggesting aminopeptidase as a potential drug target in these parasites (9).

Leucine aminopeptidases (LAPs) are found in all kingdoms of life, with many species encoding more than one LAP. They catalyze the metal-dependent hydrolysis of the N-terminal amino acid of peptide or amino acyl substrates (EC 3.4.11.1, EC 3.4.11.10, EC 3.4.11.5, and EC 3.4.11.23). The structures of LAPs from many different species have been solved (10–13), and all are homologous with overall root mean square deviations (RMSDs) between 0.27 and 0.35 Å and a sequence identity to the TriTryp LAPs of 32 to 45%. LAPs of the M17 family of aminopeptidases are bilobal proteins with a highly conserved C-terminal domain harboring the aminopeptidase activity. They form homohexamers of approximately 330 kDa with the active sites facing a central cavity separated from the bulk solvent but accessible through solvent channels. The aminopeptidase mechanism of LAPs is well characterized (14), with the activity being reported to be dependent on the presence of two cocatalytic zinc ions, although activity was shown for other divalent metal ions, e.g., manganese and magnesium (15). In contrast, the biological functions are diverse and less well understood. In bacteria, LAPs were shown to be involved in site-specific homologous recombination and transcription regulation (16, 17); in plants, LAPs moonlight as molecular chaperones independently of the aminopeptidase activity (18); in mammalian cells, they are implicated in the N-terminal processing of certain proteins (19) and have been proposed to have a role in the determination of cell redox status (20); while in some parasites, they participate in host cell invasion and the provision of amino acids essential for growth (21, 22).

There are four distinct LAPs in the TriTryp genomes, which can be classified according to their theoretical isoelectric points as acidic (LAP-A and LAP-A2), neutral (LAP-N), and basic (LAP-B). The sequence identity among the paralogues LAP-A, LAP-N, and LAP-B within a species is low, with the C-terminal domain and active site being more conserved. However, inspection of the *lap-A2* genes reveals what appears to be an incomplete LAP in all three species and their genome surroundings might suggest a gene duplication forming a nonfunctional pseudogene. The subcellular localization of each of the paralogues has been predicted with PSORT II (23) to be different, indicating distinct functions. The biochemical characterization of TriTryp LAPs to date is limited; the enzymes studied include LAP-A from *T. cruzi* (24) and LAP-B from *L. major*, *L. donovani*, *L. amazonensis* (25), and *Trypanosoma brucei*, where it has recently been shown to be required for efficient kinetoplast DNA (kDNA) division (26).

This work focused on the LAP-As of *L. major* (*Lm*LAP-A), *T. brucei* (*Tb*LAP-A), and *T. cruzi* (*Tc*LAP-A). We report the X-ray structures of *Tb*LAP-A in the metal-free (apo) and unliganded (metal-containing, holo) forms and in complex with the inhibitors bestatin and actinonin, as well as the structures of holo *Tc*LAP-A and *Lm*LAP-A in complex with actinonin. In addition, we discuss the requirements of LAP-A for survival in *T. brucei* by using RNA interference (RNAi), double knockout (dKO), and overexpression of the protein in bloodstream form (BF) and procyclic form (PF) *T. brucei* subsp. *brucei* parasites in culture. Finally, *in vivo* experiments with mice were carried out to investigate the effect of LAP-A knockdown on parasite infectivity.

## RESULTS

### Expression, purification, and confirmation of the proteolytic activity of the LAP-As.

The TriTryp LAP-As were expressed in *Escherichia coli* and purified successfully by affinity and size exclusion chromatography (SEC). The proteolytic activities of the purified enzymes against the fluorescent substrate analogues Leu-l-leucine-(4-methyl-7-coumarinylamide) (Leu-AMC), l-valine-7-amido-4-methylcoumarin (Val-AMC), and l-proline-7-amido-4-methylcoumarin (Pro-AMC) were confirmed by native gel zymography (see Fig. S1 in the supplemental material). All three LAP-As were active against Leu-AMC, while little or no activity against Val-AMC and Pro-AMC was detected. These findings are in agreement with the expected preference for substrates containing leucine as the N-terminal residue, as previously reported for this family of aminopeptidases (27).

10.1128/mSphere.00226-17.1 FIG S1  Native gel zymography of recombinant TriTryp LAP-As. Shown are native gel pieces containing the LAP-As, which were incubated with Leu-AMC, Val-AMC, or Pro-AMC for 10 min at 37°C. (A) Visualization of the gel pieces with UV light, showing the AMC products hydrolyzed by the LAP-As. (B) Coomassie staining of the same native gel pieces after (UV imaging). Tb, *T. brucei*; Tc, *T. cruzi*; Lm, *L. major*. Download FIG S1, TIF file, 2.4 MB. Copyright © 2017 Timm et al. 2017 Timm et al. This content is distributed under the terms of the Creative Commons Attribution 4.0 International license.

### Crystallization of the LAP-As.

*Tb*LAP-A crystals in space group P2_1_2_1_2_1_ with six chains in the asymmetric unit grew under citrate-containing conditions and comprise apo *Tb*LAP-A with no metal ions in the active site. *Tb*LAP-A complexes crystallized under many different conditions, preferentially in space group P2_1_ with 6 or 12 chains per asymmetric unit. All attempts to cryoprotect the crystals prior to vitrification resulted in substantial loss of diffraction quality, and only the apo *Tb*LAP-A crystals were successfully cryoprotected. *Tc*LAP-A crystallized only under citrate-containing conditions, resulting in crystals in space group R32 with one or both metal ions missing from the active site. None of the cocrystallizations with ligands yielded diffracting crystals. *Lm*LAP-A crystallized under many different conditions, forming hexagonal plate-like crystals, one of which, grown in the presence of actinonin, diffracted to 2.5 Å and was in space group P321. The crystallization conditions leading to structure solutions are summarized in Table S1.

10.1128/mSphere.00226-17.5 TABLE S1  Crystallization conditions of the LAP-As and ligands bound. Download TABLE S1, DOCX file, 0.01 MB. Copyright © 2017 Timm et al. 2017 Timm et al. This content is distributed under the terms of the Creative Commons Attribution 4.0 International license.

### The TriTryp LAP-A structures.

The TriTryp LAP-A structures were solved by molecular replacement (MR), and the crystallographic data and statistics are summarized in Tables S2 to S5. The structures show a close structural similarity to each other and LAPs of other species in the Protein Data Bank (PDB). Their nearly identical core structures have overall RMSDs for *Tc*LAP-A and *Lm*LAP-A toward *Tb*LAP-A of 0.31 and 0.33 Å, respectively, with differences limited to loop regions and minor changes in the length of secondary structure elements.

10.1128/mSphere.00226-17.6 TABLE S2  Crystallographic data and statistics for *Tb*LAP-A crystals. In this and all subsequent supplemental tables, data for the highest-resolution shell are in parentheses, the Ramachandran plot was calculated with Coot (50), and the poor rotamer analysis was carried out in MolProbity (52). Download TABLE S2, DOCX file, 0.02 MB. Copyright © 2017 Timm et al. 2017 Timm et al. This content is distributed under the terms of the Creative Commons Attribution 4.0 International license.

10.1128/mSphere.00226-17.7 TABLE S3  Crystallographic data and statistics for *Tb*LAP-A complex crystals. Download TABLE S3, DOCX file, 0.02 MB. Copyright © 2017 Timm et al. 2017 Timm et al. This content is distributed under the terms of the Creative Commons Attribution 4.0 International license.

10.1128/mSphere.00226-17.8 TABLE S4  Crystallographic data and statistics for *Tc*LAP-A crystals. Download TABLE S4, DOCX file, 0.01 MB. Copyright © 2017 Timm et al. 2017 Timm et al. This content is distributed under the terms of the Creative Commons Attribution 4.0 International license.

10.1128/mSphere.00226-17.9 TABLE S5  Crystallographic data and statistics for *Lm*LAP-A–Mn–actinonin. Download TABLE S5, DOCX file, 0.01 MB. Copyright © 2017 Timm et al. 2017 Timm et al. This content is distributed under the terms of the Creative Commons Attribution 4.0 International license.

### Overall and quaternary structures in the crystals.

**FIG 1:**
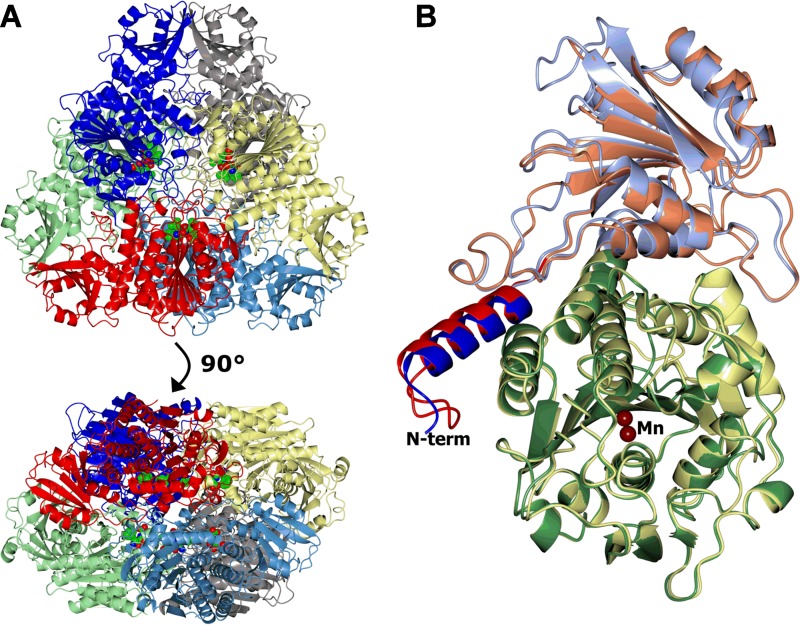
Structures of the LAP-As. Protein chains are shown in a ribbon representation. (A) Hexamer of the *Tb*LAP-A--bestatin complex colored by chain. The six bestatin ligands and associated metals are shown as spheres and lie on the inside of the hexamer. (B) Superposition of *Tb*LAP-A and *Lm*LAP-A protomers. The *Tb*LAP-A N-terminal helix (α1) is blue, the N-terminal domain is light blue, and the C-terminal domain is green. For *Lm*LAP-A, α1 is red, the N-terminal domain is coral, and the C-terminal domain is lemon. The Mn^2+^ ions in the active sites are shown as tan spheres. This and subsequent structure images were made in *CCP*4*mg* (63), and superpositions were carried out with SSM (64).

The structures of all three LAP-As show the typical hexameric architecture of the M17 metallopeptidases (Fig. 1A), and analysis with PISA (28) supports the hexamer being the stable oligomeric state in solution. All of the conformational differences seen between the TriTryp LAP-As are located at the surface of the hexamer, away from the active site or protomer-protomer interfaces.

Like other M17 family LAP structures in the PDB, the TriTryp LAP-As have two domains (N and C terminal) (Fig. 1B). The smaller N-terminal domain (amino acids [aa] 2 to 197) consists of a six-stranded β sheet (β1, β6, β5, β2, β4, and β3) flanked by five α helices (α2 to α6). Unlike other LAPs in the PDB, TriTryp LAP-As have an α helix at the N terminus (α-1) that interacts with the neighboring protomer of the hexamer. The N-terminal domain is connected to the C-terminal domain via a long α helix (α7). The highly conserved C-terminal domain (aa 215 to 521), nearly identical to the structures of other LAPs in the PDB, shows a central eight-stranded β sheet (β7, β8, β10, β9, β13, β17, β14, and β15) surrounded by α helices (α8 to α17) and a small β sheet (β11, β12, and β16). The C-terminal domain contains the active site and forms the core of the hexamer, arranged around a central 3-fold axis with the active sites facing the central cavity, secluded from the surrounding bulk solvent.

In *Tc*LAP-A, some loop regions in the N-terminal domain (between α2 and β2, β3 and β4, α5 and β5, and α6 and β6) and in the C-terminal domain (between β8 and β9 and between β10 and α11) show conformations slightly different from those in *Tb*LAP-A.

Structural differences in *Lm*LAP-A compared to *Tb*LAP-A and *Tc*LAP-A are mostly located in the loop regions. The secondary structure elements and their positions in *Lm*LAP-A are nearly identical to those in *Tb*LAP-A, with the exceptions of β1, β3, and β4, which are shortened, and α2, which is replaced by a loop. The N terminus of *Lm*LAP-A is extended and forms a loop before the start of the α1 helix. The loop regions noted as different for *Tc*LAP-A are extended in *Lm*LAP-A and again adopt different conformations. Additionally, the loop between β6 and α7 is extended, harboring a small α helix, and helix α11 in the C-terminal domain is extended by one turn.

### Metal coordination in the active site and apo LAP-A.

**FIG 2:**
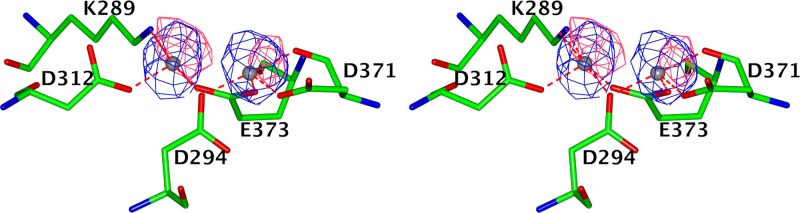
Stereo view of the metal coordination and anomalous difference density in the active site of *Tb*LAP-A. The amino acid residues coordinating the two Mn^2+^ ions in the active sites are shown as cylinders colored by atom type. The anomalous difference maps calculated from data collected on the same crystal at 0.9795 and 1.3051 Å are shown at the 4 σ level in coral and blue, respectively.

The metal-coordinating amino acid residues in the TriTryp LAP-As are conserved and identical to those in previously reported LAP structures. The two metal binding sites were shown to have different binding affinities for the metal ions, with site 1 generally having a lower affinity than site 2 (15, 29). In *Tb*LAP-A, the carboxylate oxygen atoms of D294 and E373 and the main-chain and carboxylate oxygens of D371 coordinate the metal ion in site 1 and the carboxylate oxygens of D294, E373, and D312 and the ε-amino group of K289 coordinate that in site 2 (Fig. 2). The corresponding residue numbers in *Tc*LAP-A are K288, D293, D311, D370, and E372, and those in *Lm*LAP-A are K299, D304, D322, D389, and E391.

The holo *Tb*LAP-A (crystallized in the presence of leucine), *Tb*LAP-A complexes, and *Lm*LAP-A structures have two metals bound in the active site; the *Tc*LAP-A structures, in contrast, clearly show only one metal ion in site 1 and no metal in site 2. However, the active-site residues adopt the same conformations as if two metal ions were bound.

Most LAP-A crystals were grown in the presence of manganese ions. As LAPs are reported to be zinc-dependent enzymes, crystals of EDTA-treated *Tb*LAP-A were grown in the presence of ZnCl_2_. Nevertheless, X-ray fluorescence (XRF) scans of the resulting crystals indicated the presence of manganese ions, suggesting that EDTA may not to be able to efficiently chelate the Mn^2+^ ions at the concentrations used. A second data set was collected from the same crystal at a wavelength of 1.3052 Å, between the absorption edges of manganese and zinc, and an anomalous difference Fourier synthesis was calculated for the data sets at both wavelengths by using phases from the refined model (Fig. 2). The map for the 1.3052-Å wavelength data showed peaks at around 10 σ for both metal sites, substantially larger than the 7 σ peaks for the original data recorded with a wavelength of 0.97949 Å, clearly indicating that neither site was occupied by zinc. The metals were therefore modeled as manganese ions.

The structures of LAP-As in their apo and holo forms and in complex with inhibitors are practically identical. The exception is the metal-free apo *Tb*LAP-A form, whose active-site loop (aa 293 to 305) is disordered and in which no electron density for bound metal ions could be detected, most likely because of the high concentration of citrate in the crystallization buffer. Apo *Tc*LAP-A and the other LAP-A structures show completely ordered active sites independent of metal or ligand binding.

### The *Tb*LAP-A--bestatin complex.

**FIG 3:**
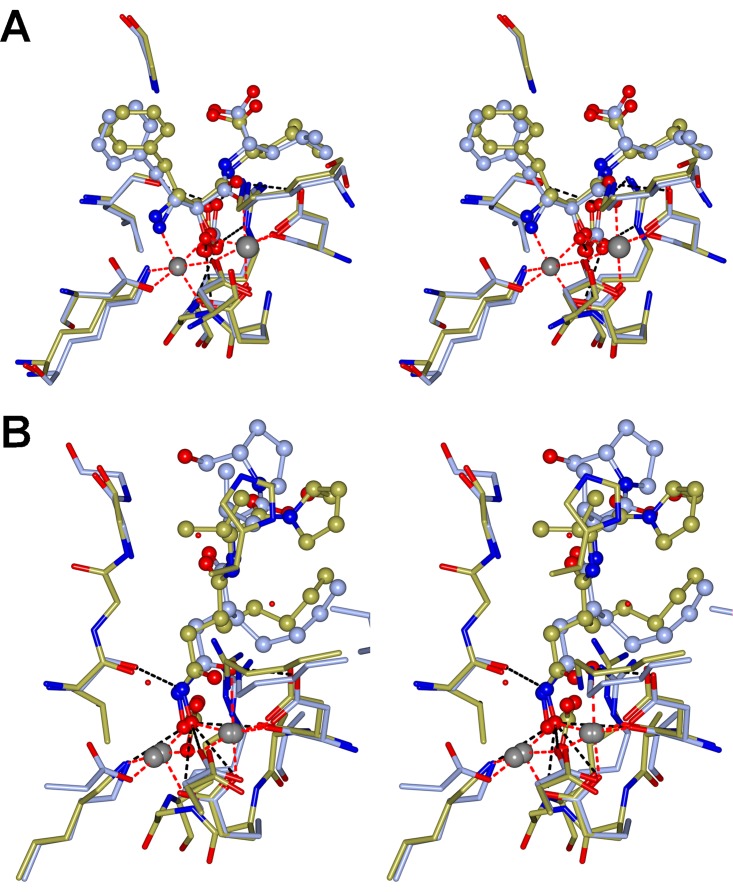
Inhibitor binding in the active site of the LAP-As. (A) Stereo images of the active site of *Tb*LAP-A in complex with Mn^2+^ and bestatin superposed on the active site of the *Pf*LAP-bestatin complex (PDB code 3KR4). The coordinating residues are shown as cylinders, and the ligand is shown in a ball-and-stick representation, both colored by atom type with the C in ice blue (*Tb*LAP-A) or gold (*Pf*LAP-A). The metal coordination is represented by red dashed lines, and hydrogen bonds are shown as black dashed lines. (B) Stereo images of the active sites of *Tb*LAP-A and *Lm*LAP-A in complex with Mn^2+^ and actinonin. Amino acid residues and ligands are colored by atom type, with the C in ice blue (*Tb*LAP-A) or gold (*Lm*LAP-A). In both, the proteins were first superposed by using the SSM option in *CCP*4*mg* for residues from the C-terminal domain (300 to 500), followed by the “match close residues” option.

*Tb*LAP-A was cocrystallized with the inhibitor bestatin. The electron density of the ligand was well defined in all 12 chains in the asymmetric unit and was modeled in the same conformation (Fig. 3A). The bestatin conformation mimics the *gem*-diolate intermediate of the peptide substrate in the process of its cleavage, and its binding in *Tb*LAP-A is identical to that in *Pf*LAP (PDB code 3KR4). The coordination of bestatin is mainly facilitated through the Mn^2+^ ions and hydrogen bonding. The Mn^2+^ ions coordinate the N-2 nitrogen atom and O-2 and O-3 oxygen atoms. Additionally, N-2 is coordinated through hydrogen bonds with the side chain oxygens of D294 and D312, as well as the main-chain oxygen of T401. The main-chain oxygen of D371 and the side chains of K289 and D294 form hydrogen bonds with O-2, while the side chain of K301 forms a hydrogen bond with O-3. Furthermore, bound HCO_3_^−^ forms a hydrogen bond with the O-2 atom. While hydrophobic residues surround the phenyl ring of bestatin, its carboxyl group forms hydrogen bonds with G404 and R467.

### The actinonin complexes.

For both the *Tb*LAP-A and *Lm*LAP-A complexes, the electron density was sufficient to model the ligand, albeit with less than 100% occupancy. The limited resolution and quality of the data sets do not allow accurate determination of the ligand’s conformation. The *Tb*LAP-A--actinonin complex had six molecules in the asymmetric unit, and local noncrystallography symmetry was imposed for the protein chains. The electron density for the ligand was generally poor but sufficient to build the actinonin in all six chains. Their densities varied slightly, so that the ligand was modeled in each chain individually, with variation in their conformations. Nevertheless, the actinonin coordination was identical in all chains and differed only slightly from that in *Lm*LAP-A (Fig. 3B).

Actinonin in *Lm*LAP-A and *Tb*LAP-A is coordinated by the Mn^2+^ ions and hydrogen bonds. In both structures, the two Mn^2+^ atoms coordinate the O-2 and O-4 oxygen atoms of the ligand. In *Lm*LAP-A, the side chains of K299, D304, K311, and D389 form hydrogen bonds with O-2 and O-4, and the main-chain oxygen of L420 and the main-chain nitrogen of G422 form hydrogen bonds with N-1 and O-13, respectively. The N-14 atom of actinonin coordinates a water molecule. The corresponding residues in *Tb*LAP-A are K289, D294, K301, D371, L402, and G404, and A405 forms an additional hydrogen bond with O-27 in four of the six chains. Because of the limited data quality, no water molecules were modeled in the *Tb*LAP-A structure, with the exception of those replacing the HCO_3_^−^ molecule. Superposition of the active sites including the actinonin molecules reveals the high similarity of the conformations actinonin adopts, with the only difference being in the hydroxymethyl-pyrrolidine moiety, which forms a hydrogen bond in *Tb*LAP-A but not in *Lm*LAP-A, where it is bent away from the corresponding residue (A424).

### *Tc*LAP-A in complex with citrate.

*Tc*LAP-A crystallized exclusively under citrate-containing conditions, which led to the absence of one or both Mn^2+^ ions from the active site and hence no electron density for the inhibitors. The structure obtained from cocrystallization with the inhibitor amastatin showed clear density for one partially occupied Mn^2+^ ion and a citrate molecule bound in the active site. There was no electron density for amastatin in this structure, and HCO_3_^−^ is replaced by SO_4_^2−^ (Fig. S2). The citrate ion is coordinated by the Mn^2+^ and hydrogen bonding, with Mn^2+^ coordinating the O-3 and O-4 atoms from citrate, K288 forming a hydrogen bond with O-4, and K300 forming hydrogen bonds with O-3 and O-5. Atom O-7 from the citrate forms hydrogen bonds with D311, M308, and a water molecule. Main and side chains of R374, G373, and K288 coordinate the SO_4_^2−^ molecule.

10.1128/mSphere.00226-17.2 FIG S2  Citrate binding in *Tc*LAP-A. Shown is a stereo image of the active-site residues including the Mn^2+^, SO_4_^2−^, and citrate (CIT) coordination. Citrate, SO_4_^2−^, and coordinating side chains are shown as cylinders colored by atom type, with the side chain C atoms in orange and Mn^2+^ as a gray sphere. The white sphere indicates the location of the empty metal binding site. The metal coordination and hydrogen bonds are drawn as red and black dashed lines, respectively. Download FIG S2, TIF file, 1 MB. Copyright © 2017 Timm et al. 2017 Timm et al. This content is distributed under the terms of the Creative Commons Attribution 4.0 International license.

### Oligomeric state in solution.

The LAP-As crystallized as hexamers, as reported for other LAP structures in the PDB. To confirm the hexamer as the oligomeric state in solution, SEC-multiangle laser light scattering (SEC-MALLS) analyses of all three LAP-As were carried out at a range of protein concentrations. The expected molecular masses for the different oligomeric states are summarized in Table S6 and were used to annotate the elution peaks obtained from the SEC-MALLS data allowing for an error of ±10%. An overlay of the differential refractive index traces at a protein concentration of 1.0 mg·ml^−1^ with annotated peaks according to the oligomeric states corresponding to the molecular masses is shown in Fig. S3. The analysis of *Tc*LAP-A showed the presence of two well-separated peaks, one with a calculated molecular mass of ~320 kDa and the other with a mass of ~170 kDa, corresponding to the hexamer and trimer, respectively. At all of the concentrations analyzed, the hexamer was the most abundant species, while as the concentration was lowered, the proportion of trimers increased. Unlike for *Tb*LAP-A, the hexameric and trimeric species were separated well on the column, which suggests a slower interconversion of the hexamer and trimeric *Tc*LAP-A species than in *Tb*LAP-A. Analysis of *Lm*LAP-A showed three distinct molecular mass species, corresponding to the hexamer, trimer, and monomer, plus a considerable amount of aggregation. The three species were well separated from each other and the aggregates in the void volume. The lower the protein concentration, the greater is the proportion of monomers. Whether the monomers are a result of instability and subsequently result in their aggregation or if the monomeric state is physiologically relevant remains unclear and requires further investigation.

10.1128/mSphere.00226-17.10 TABLE S6  Expected molecular masses of different LAP-A oligomers. Download TABLE S6, DOCX file, 0.01 MB. Copyright © 2017 Timm et al. 2017 Timm et al. This content is distributed under the terms of the Creative Commons Attribution 4.0 International license.

10.1128/mSphere.00226-17.3 FIG S3  Annotated SEC-MALLS data for *Tb*LAP-A, *Tc*LAP-A, and *Lm*LAP-A. Shown are the overlaid differential refractive index traces of the three LAP-As each at 1 mg·ml^−1^. Download FIG S3, TIF file, 2.5 MB. Copyright © 2017 Timm et al. 2017 Timm et al. This content is distributed under the terms of the Creative Commons Attribution 4.0 International license.

### Knockdown and knockout of LAP-A do not impair *T. brucei* proliferation.

To explore the potential of *Tb*LAP-A as a drug target and assess its essentiality for *T. brucei* survival, the expression of the enzyme in both BF and PF *T. brucei* cells was knocked down by using an RNAi stem-loop/stuffer strategy. This created trypanosome lines that expressed a 465-bp fragment of the *TbLAP*-A gene under the control of a tetracycline-inducible promoter.

**FIG 4:**
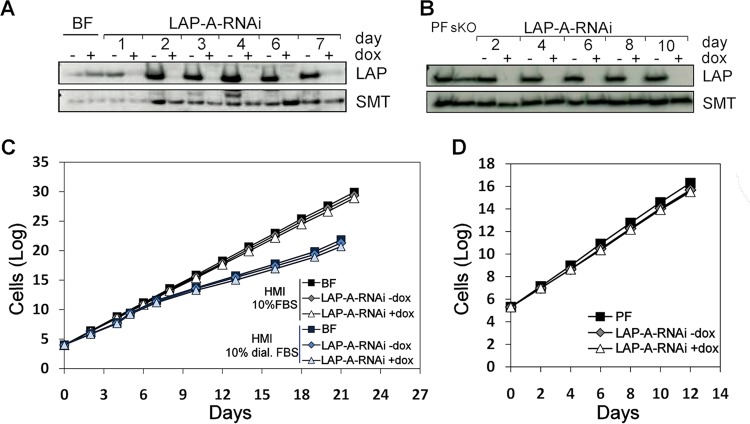
RNAi-mediated depletion of LAP-A in PF and BF parasites. The effect of RNAi-mediated depletion of LAP-A on BF (C) and PF (D) parasites was analyzed. Western blot analyses of LAP-A-RNAi BF (A) and LAP-A-RNAi PFs (B) parasites at different time points are shown. In Western blot analyses, anti-*Tb*LAP-A antibody was used at a 1:15,000 dilution; the loading control was rabbit anti-*Tb*SMT antibody at a 1:10,000 dilution. Each lane contained 2.5× 10^6^ parasites. Cumulative growth curves of LAP-A RNAi BF *T. brucei* analyzed in both HMI-9 medium containing FBS or dialyzed FBS (C) and LAP-A RNAi PF (D) are shown. dox, doxycycline.

**FIG 5:**
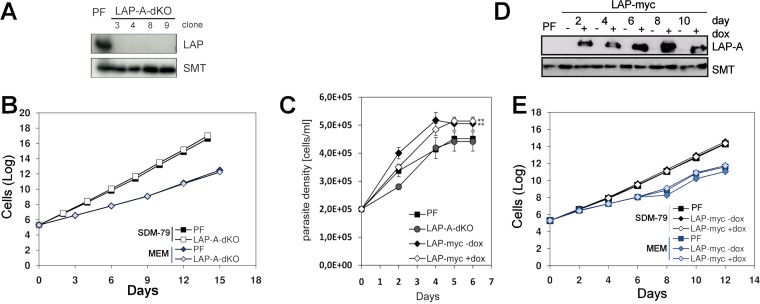
Growth phenotype of LAP-A-dKO and LAP-A-OE cells in different media and effect of leucine depletion. dKO mutants were obtained with PFs (LAP-A-dKO), and cell proliferation was assessed. (A) Western blot analysis indicating successful elimination of LAP-A expression in different cell clones. (B) Cumulative growth curves of PF *T. brucei* cultured in SDM-79 containing FBS and MEM containing dialyzed FBS. (C) Cumulative growth curves of parental, *Tb*LAP-A-OE (LAP-myc), and LAP-A-dKO cells grown in MEM devoid of leucine. (D) Western blot analysis indicating the level of expression of LAP-A at different time points. Cells were diluted every 48 h. For Western blot analyses, anti-*Tb*LAP-A antibody was used at a 1:15,000 dilution; the loading control was rabbit anti-*Tb*SMT antibody at a 1:10,000 dilution. Each lane contained 2.5× 10^6^ parasites. (E) Cumulative growth curves of LAP-A-OE PF parasites grown in SDM-79 and glucose-free MEM. dox, doxycycline.

*TbLAP*-A gene knockdown in BF and PF parasites was monitored by Western blotting (Fig. 4A and B, respectively), and the resulting phenotype was examined in different media. The RNAi-mediated depletion of LAP-A approached 95 and 100% in BF and PF parasites, respectively, and was maintained throughout the period studied. With regard to the effect of LAP-A depletion on parasite proliferation, RNAi-depleted strains of BF parasites cultured in HMI-9 medium showed a growth rate similar to that of controls (Fig. 4C). When dialyzed fetal bovine serum (FBS) was used, the growth rate was slightly diminished yet similar in both control and LAP-A-depleted cells (Fig. 4C). For PF parasites, RNAi-mediated knockdown of the enzyme did not result in significant growth defects in SDM-79 medium (Fig. 4D). In addition, a LAP-A knockout strain was achieved for PF cells. Efficient elimination of enzyme expression is shown in Fig. 5A. In minimal essential medium (MEM) containing dialyzed FBS and therefore devoid of glucose, a growth rate reduction of ~20% was observed in both control and dKO parasites (Fig. 5B). In summary, LAP-A is not an essential enzyme for the *in vitro* growth of either form of the *T. brucei* subsp. *brucei* parasite when it is cultured in a range of media.

10.1128/mSphere.00226-17.4 FIG S4  Anti-*Tb*LAP-A antibody specificity. Western blot analysis of PF *T. brucei* whole-cell extract (WCE) hybridized with anti-*Tb*LAP-A antibody (1:100,000). The values to the left are molecular sizes in kilodaltons. Download FIG S4, TIF file, 1.1 MB. Copyright © 2017 Timm et al. 2017 Timm et al. This content is distributed under the terms of the Creative Commons Attribution 4.0 International license.

### Role of LAP-A in leucine starvation.

LAPs cleave amino acids, preferentially leucine, from the N terminus of proteins or peptides and are important for protein recycling and turnover. It is reasonable to speculate that the depletion or overexpression of an aminopeptidase will have an increased effect on cell fitness upon exposure to suboptimal extracellular amino acid concentrations. In the event of LAP-A depletion, extracellular leucine levels might be particularly important. To test the effect of leucine limitation on the growth and cell cycle of PFs of *T. brucei* subsp. *brucei*, cultures were grown in glucose-free MEM prepared without leucine and with dialyzed FBS to ensure that no small peptides or undefined amino acids were present. Parental and LAP-A-dKO cells from cultures grown in MEM containing leucine (with dialyzed FBS) were transferred to MEM without leucine and incubated at culture temperature. In the absence of leucine, both the parental and LAP-A-dKO parasites grew deficiently (Fig. 5C).

Additionally, to provide an insight into the function of LAP-A in trypanosomes, overexpressing cell lines were generated and the resulting phenotype was analyzed. Overexpression of LAP-A fused to a myc tag in both BF and PF parasites was induced by the addition of doxycycline. LAP-A overexpression in BFs was minor (~1.2-fold) and had no significant effect on proliferation, while LAP-A-overexpressing (LAP-A-OE) PF parasites showed significantly increased levels of LAP-A (2.5- to 3-fold), as assessed by Western blotting (Fig. 5D), yet grew at a rate similar to that of controls (Fig. 5E). Overexpressing PFs were further grown in MEM containing dialyzed FBS to monitor whether the presence of high levels of the enzyme confers a selective advantage under conditions of restricted nutrients. Lower growth rates were obtained with all of the cell lines tested compared to SDM-79, and LAP-A overexpression appeared to be slightly detrimental under these conditions (Fig. 5E). However, when cultured in glucose-free MEM without leucine, LAP-A-OE parasites grew at a higher rate than controls (Fig. 5C).

### Subcellular localization of LAP-A in *T. brucei*.

**FIG 6:**
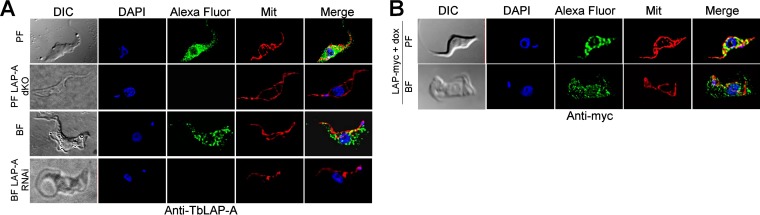
*Tb*LAP intracellular localization. (A) Immunofluorescence analysis of the intracellular localization of *Tb*LAP in parental PF and BF parasites with anti-*Tb*LAP primary antibody and an Alexa Fluor 488-conjugated anti-rabbit antibody. RNAi-depleted BF parasites after induction for 4 days and LAP-A-dKO PF parasites are also shown. Additional immunofluorescence studies were performed with both BF and PF parasites overexpressing *Tb*LAP fused with a myc tag (BF and PF LAP-myc) with an anti-myc monoclonal antibody and an Alexa Fluor 488-conjugated goat anti-mouse antibody (B). *Tb*LAP and *Tb*LAP-myc are green. Mitochondria stained with MitoTracker Red CMXRos (Mit) and nuclei and kinetoplasts stained with DAPI are red and blue, respectively. Images were collected with an Olympus IX81 microscope and deconvolved with Huygens Professional from Scientific Volume Imaging (version 3.3) and pseudocolored with Fiji software (61). DIC, differential interference contrast.

BFs and PFs of *T. brucei* were grown to mid-log phase and fixed on poly-l-lysine-coated glass slides for determination of the intracellular localization of *Tb*LAP-A. Mitochondria were stained with fluorescent MitoTracker Red, and nuclear DNA and kDNA were visualized with 4',6-diamidino-2-phenylindole (DAPI). *Tb*LAP-A was detected with affinity-purified rabbit anti-*Tb*LAP-A antiserum. Cells were imaged by confocal and wide-field fluorescence microscopy (Fig. 6). Parasites overexpressing c-Myc-tagged LAP-A (2 days of induction) were also analyzed with a c-Myc-specific antibody. c-Myc-tagged LAP-A exhibited a cytosolic distribution similar to that obtained with the anti-*Tb*LAP-A antibody in the parental cell line. Colocalization analysis indicated a minor overlap with mitochondria (Pearson’s coefficient, 0.5 ± 0.1; Manders’ overlap coefficient, 0.27 ± 0.07). Thus, while *Tb*LAP-A is mainly cytosolic, a minor level within the mitochondrion in BFs and PFs cannot be fully excluded. The staining disappeared upon RNAi-mediated knockdown and in LAP-A-dKO cells, validating the specificity of the polyclonal antibody and the efficacy of RNAi-mediated depletion (Fig. 6).

### Effect of nutritional starvation on LAP-A subcellular localization.

**FIG 7:**
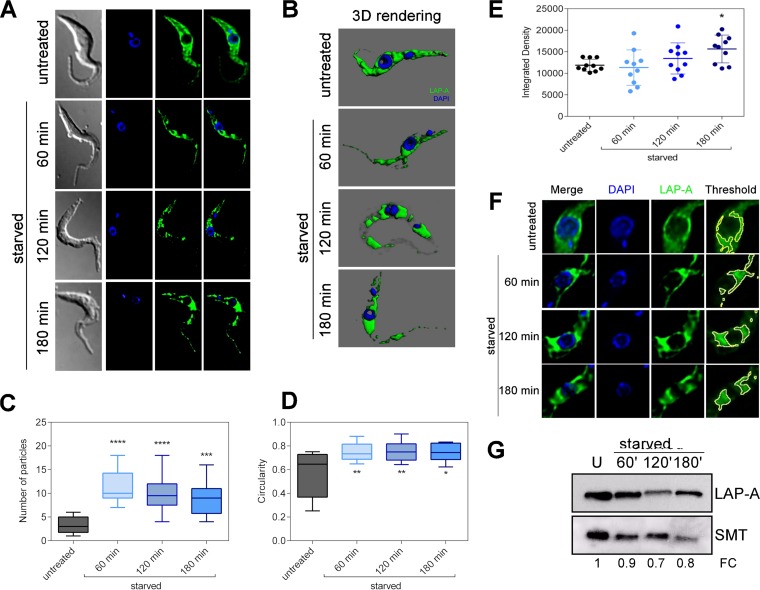
*Tb*LAP-A intracellular localization during nutritional starvation. (A) Immunofluorescence analysis of *Tb*LAP intracellular localization in parental PFs under normal conditions (untreated) or during 60, 120, or 180 min of nutritional starvation (starved). (B) Three-dimensional rendering of immunofluorescent parasites showing LAP-A localization during nutritional stress performed with Imaris 4.0.6 Bitplane AG. (C) Number of particles of LAP-A signal in untreated or starved PF parasites obtained with Fiji Particle Analyzer (ImageJ 1.51d) (61) by using the automatic default threshold; the box represents the interquartile range, and the whiskers are maximum and minimum values. (D) Shape analysis of LAP-A particles represented by the circularity factor obtained with Fiji Particle Analyzer (ImageJ 1.51d) (61) by using the automatic default threshold. (E) Integrated density analysis of LAP-A signal in the perinuclear area obtained with Fiji Particle Analyzer (ImageJ 1.51d) by using the manual threshold (9.1% ± 0.1%). (F) Detail of the perinuclear area in untreated and starved parasites. The example threshold is also shown. The boxes represent the interquartile range, and the whiskers are maximum and minimum values. (G) Western blot analysis of PF whole-cell extracts during nutritional starvation. U, untreated. Fold changes (FC) in LAP-A expression calculated with respect to unstarved parasites are shown. Asterisks represent *P* values calculated by two-way analysis of variance with respect to untreated parasites. ****, *P* < 0.0001; ***, *P* < 0.0001; **, *P* < 0.001; *, *P* < 0.01.

Under stress conditions such as nutritional starvation, eukaryotic cells activate several mechanisms to recycle the cellular content, which may cause changes in the localization of the enzymes involved in these processes. Considering the potential participation of LAP-A in protein recycling and amino acid supply, we sought to examine the effect of nutritional starvation on its subcellular localization and expression. *T. brucei* PF parasites were starved for 60, 120, or 180 min by incubation in phosphate-buffered saline (PBS) and then subjected to immunofluorescence analysis with the anti-*Tb*LAP-A antibody. Under normal conditions (untreated cells), LAP-A is homogeneously spread all over the cytoplasm (Fig. 7A and B). However, upon nutritional starvation, the enzyme progressively relocalized and accumulated in specific regions, especially in the cytoplasmic perinuclear area (Fig. 7A and B). This phenomenon was quantified by determining the number of particles containing LAP-A in each cell while applying an automatic threshold. In untreated cells, LAP was present at an average of three particles per cell, one of which corresponds to almost the entire cytoplasm, while during starvation, the number of particles significantly increased (Fig. 7C). The shape of the particles resulting from LAP-A accumulation in foci during starvation was also analyzed by measuring circularity, which corresponds to 1 in the case of a perfect circle and 0 in the case of an infinitely elongated polygon. LAP-A-containing particles were significantly more circular in starved cells than in untreated cells (Fig. 7D). Finally, quantification of the LAP-A signal intensity in the perinuclear region showed that significant accumulation of the protein occurs in this area during PBS treatment (Fig. 7E and F). However, the total expression of *Tb*LAP-A remains almost unchanged or slightly decreases during starvation (Fig. 7G). In mammalian cells, it has been reported that lysosomes and autophagic vesicles cluster in the perinuclear area, driven by intracellular pH changes in response to nutritional starvation. Likewise, a perinuclear localization of autophagosomes has been observed in trypanosomatids. Thus, *Tb*LAP-A relocalization during nutritional starvation suggests a possible involvement of *Tb*LAP-A in protein degradation that occurs in response to nutrient restriction.

### Importance of LAP-A *in vivo*.

**FIG 8:**
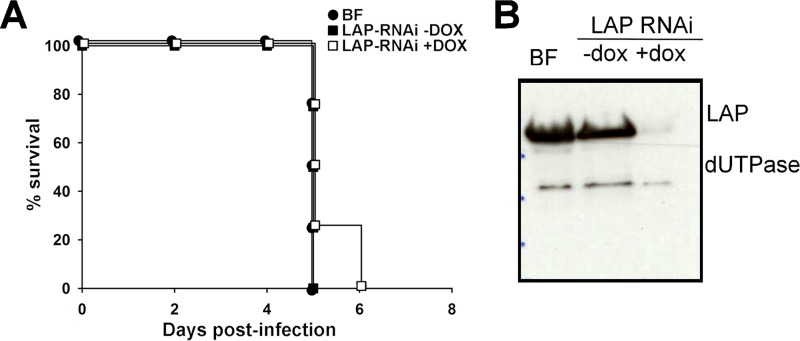
Survival profile analysis of mice infected with *Tb*LAP-A-depleted T. brucei subsp. *brucei*. (A) Kaplan-Meier survival analysis of C57BL/6J mice infected with the parental BF and *Tb*LAP-A RNAi lines in the absence or presence of doxycycline (DOX) to induce *Tb*LAP-A knockdown. (B) Western blot analysis of parasites recovered from infected animals showing efficient RNAi-mediated depletion of LAP-A.

To evaluate the role of *Tb*LAP-A in parasite survival within the mammalian host, C57BL/6J mice were infected with the *Tb*LAP-A RNAi and parental strains, downregulation *in vivo* was induced by doxycycline administration, and the progression of the infection was monitored. After a few days, both the parental and *Tb*LAP-A RNAi cell lines showed high levels of parasitemia (10^9^ parasites ml^−1^) in mice that consequently resulted in death within 5 to 6 days postinfection (Fig. 8A), indicating that *Tb*LAP-A is not essential for parasite viability *in vivo*. Efficient depletion of the enzyme *in vivo* was monitored by Western blotting (Fig. 8B).

## DISCUSSION

### LAP-A as a potential drug target in *T. brucei*.

The data obtained indicate that LAP-A is not essential for survival and proliferation of BF and PF *T. brucei* subsp. *brucei* parasites under the conditions tested either *in vitro* or *in vivo*. RNAi-mediated depletion in BF parasites and both RNAi-mediated depletion and the generation of dKO mutants in PFs support this conclusion. Whether this is the result of redundancy caused by the presence of additional M17 LAPs, paralogues of the one investigated, requires further research. Recent evidence obtained with other parasites also questions the essential character of LAP-A. Thus, bestatin, a broad-spectrum metalloaminopeptidase inhibitor (30) reported to inhibit the M17 LAP of *T. cruzi* (24), was not cytotoxic against *T. cruzi* after treatment at 300 μM for several hours (31) under conditions where multiple dipeptides were shown to be increased, confirming *in situ* inhibition of the LAP. In addition, a study with *Toxoplasma gondii* has shown that LAP knockout has a moderate effect on growth but does efficiently block development or virulence, suggesting that it is inadequate as a drug target also in this parasite (32). Conversely, *Tb*LAP-B has been recently shown to be required for correct segregation of kDNA, with knockdown resulting in delayed cytokinesis and growth defects (26).

The observation that LAP-A is not an essential protein in *T. brucei* contrasts with its function in *P. falciparum*, where its drug target potential has been demonstrated (33, 34). Likewise, it does not have an important role in survival in the mammalian host since the infectivity of RNAi-depleted parasites in mice was similar to that of controls. Because of the profound differences in metabolism and life cycle stages among the TriTryps, the results obtained with *T. brucei* subsp. *brucei* parasites do not allow conclusions to be drawn about LAP-A’s potential as a drug target in the intracellular parasites *T. cruzi* and *L. major*, where it may be involved in host cell invasion.

*Tb*LAP-N is predicted to localize to the endoplasmic reticulum by PSORT II (23), while *Tb*LAP-B has been shown to localize to the kinetoplast (26) and has a function distinct from that of LAP-A, which is cytosolic. The establishment of subcellular LAP-A as a cytosolic enzyme may contribute to the understanding of its physiological function, which would be distinct from the potential endoplasmic reticulum neutral aminopeptidase. Thus, while LAP-A would perform metabolic or housekeeping functions in the cytosol, in the endoplasmic reticulum, it could be involved in the posttranslational modification of N-terminal signal peptides of specific proteins, performing a regulatory role. In the present study, the enzyme appears to be mainly cytosolic and mostly excluded from the nucleus, and hence, an involvement in site-specific homologous recombination, as reported in *E. coli* (35, 36), also seems improbable. A similar localization of LAP-B was described in immunocytochemical studies with antibodies in *L. amazonensis* promastigotes, where diffuse fluorescence throughout the parasite cytosol was observed (25). While not essential for efficient proliferation, LAP-A may be involved in sensitivity to leucine and nutrient starvation. In mammalian cells, leucine starvation has been shown to induce increased lysosome-dependent protein breakdown to replenish amino acid pools (37). In *T. brucei* PF cells, starvation stress induces the presences of a functional autophagy pathway (38). PF parasites exposed to leucine depletion grew at very low rates and entered a short stationary phase before undergoing cell death. LAP-A-OE parasites appear to exhibit consistently a growth advantage under these conditions, which suggests a role for the enzyme in protein breakdown and the maintenance of leucine pools. Interestingly upon nutrient starvation, the location of LAP-A changed and concentrated in vesicles in the nuclear periphery. While the nature of these vesicles remains to be established, we postulate that LAP-A is recruited for specific functions related to protein and amino acid homeostasis and may be important for the cellular response to events related to a limited nutrient supply.

### Structure and oligomeric state of LAP-A and their effects on possible physiological substrates.

The TriTryp LAP-As form hexamers in solution and in the crystal, and homology to other LAPs indicates that this oligomeric state is a key feature of this family of enzymes. The active site of each protomer faces the inner cavity, sequestered from the bulk solvent. Although access channels allow diffusion into the inner cavity and the active sites, the accessibility seems restricted to small, unfolded substrates. This favors LAP-A’s function as a mainly metabolic enzyme whose function is housekeeping or trimming of the N termini of specific unfolded peptides. Nevertheless, the bacterial M17 family LAPs PepA and PepB were shown to cleave the loosely folded protein casein *in vitro* (39), indicating LAP’s potential to process larger substrates.

### The physiological metal ion in the active site of LAP-A.

While early studies on the bovine lens LAP showed Zn^2+^ to be the physiological metal ion in the active site of the M17 family LAPs (15), the enzyme was also shown to be active with other divalent metal ions (24, 25, 40–42). It was noted earlier (12) that *Pseudomonas putida* LAP produced in *E. coli* has Mn^2+^ in its active site, with the substrate specificity being influenced by the identity of the metal in the active site (20), with sulfhydryl-containing substrates only being accepted by Mn^2+^-containing LAPs and not by Zn^2+^-containing LAPs.

Within this study, the best crystals of the TriTryp LAP-As grew in the presence of Mn^2+^ and indeed, crystals grown under Zn^2+^-containing conditions still retained Mn^2+^ in the active site. This might indicate that Mn^2+^ is the physiological metal copurified with the enzyme expressed in *E. coli*. Given the similar estimates of manganese and zinc ion concentrations on the order of 10 to 100 μM in the cytosol, further studies are required to confirm which of these is the physiological metal in these enzymes.

In summary, the structures of the TriTryp LAP-As were solved in the apo form and several ligand complexes, which gives insight into ligand binding and confirms the homology to other family members. The biological data show that LAP-A is not essential either for growth *in vitro* or for infectivity in mouse models, therefore precluding its exploitation as a drug target. Protection against leucine depletion and the behavior of the enzyme during starvation suggest a possible role in the response to nutrient restriction.

## MATERIALS AND METHODS

### Cloning of LAP-A genes and protein expression in *E. coli*.

The genes encoding the LAP-As were amplified from genomic DNA (*T. brucei* Lister427, *T. cruzi* Sylvio X10/1, and *L. major* Friedlin) and cloned into the YSBLIC3C vector by ligase-independent cloning (43). Successful cloning was confirmed by restriction endonuclease digestion and sequencing. The resulting constructs (p*Tb*LAP-3C, p*Tc*LAP-3C, and p*Lm*LAP-3C) encoded the LAP-A proteins with an N-terminal fusion to a hexahistidine tag separated from the N terminus of the protein by the recognition site of the human rhinovirus (HRV) 3C protease. Recombinant full-length *Tb*LAP-A was overexpressed from the p*Tb*LAP-3C vector into BL21(DE3) cells grown in LB medium supplemented with 35 µg·ml^−1^ kanamycin. *Tc*LAP-A and *Lm*LAP-A recombinant proteins were expressed from the p*Tc*LAP-3C and p*Lm*LAP-3C vectors into pLEMO(DE3) cells grown in LB medium supplemented with 35 µg ml^−1^ kanamycin and 34 µg ml^−1^ chloramphenicol. *Tb*LAP-A expression was induced with 1 mM isopropyl-β-d-thiogalactopyranoside (IPTG) for 4 h at 37°C. *Tc*LAP-A and *Lm*LAP-A expression was induced overnight at 20°C with 1 mM IPTG. Cells were harvested by centrifugation, and the pellet was frozen at −20°C.

### Purification and confirmation of peptidolytic activity.

Cell pellets from each culture were thawed on ice, resuspended in buffer A (20 mM Tris [pH 8.0], 300 mM NaCl, 30 mM imidazole, 1 mM dithiothreitol [DTT]) containing 100 μM phenylmethylsulfonyl fluoride, and lysed by sonication. The cell debris was removed by centrifugation for 30 min at 5,000 × *g* and 4°C, and the supernatant was loaded onto an equilibrated 5-ml HisTrap crude FF column (GE Healthcare). Protein was eluted with an imidazole gradient of 30 to 500 mM in buffer A, and the protein-containing fractions identified by SDS-PAGE were pooled. The LAP-As with cleavable N-terminal His tags were concentrated to ~30 ml before the addition of HRV 3C protease in an approximate ratio of 1:100 (protease-to-LAP-A ratio). Proteolysis was monitored by SDS-PAGE and reached completion after 16 h at 4°C. Subsequently, the protein buffer was exchanged into buffer A with centrifuge filters (Amicon Ultra Centrifugal Filter from Millipore or Vivaspin6 from Sartorius Stedim Biotech, 50-kDa cutoff) and another nickel affinity purification performed as described above. The untagged LAP-A in the flowthrough fraction was concentrated to an ~1-ml total volume and injected onto a Superdex 200 16/600 column (GE Healthcare) equilibrated in SEC buffer (10 mM Tris [pH 8.0], 200 mM NaCl, 1 mM DTT). *Tb*LAP-A fused to a noncleavable His tag was concentrated to ~1 ml and loaded directly onto the equilibrated Superdex 200 16/600 column after the first nickel affinity purification step. The final purity was analyzed by SDS-PAGE, and the protein-containing fractions of one elution peak were pooled and buffer exchanged into a mixture of 20 mM Tris (pH 8.0), 300 mM NaCl, and 1 mM DTT. The LAP-As were concentrated, vitrified in liquid nitrogen, and stored at −80°C.

Each of the purified LAP-As was mixed with native gel loading buffer (100 mM Tris [pH 6.8], 20% [vol/vol] glycerol, 0.1% [wt/vol] bromophenol blue) and loaded three times onto one 5% polyacrylamide gel. Electrophoresis was carried out at 100 V for 2 h at 4°C to prevent denaturation of the proteins. Afterward, the gel was washed three times for 20 min in 50 mM Tris (pH 8.0) and subsequently cut into three pieces, with each piece containing all three LAP-As. Each gel piece was incubated in activity assay buffer (50 mM Tris [pH 8.0], 100 mM KCl, 1 mM MnCl_2_) containing 7.5 µM fluorescent substrate for 10 min at 37°C in the dark. The fluorescent substrates tested were Leu-AMC, Val-AMC, and Pro-AMC. After incubation, the fluorescent products on the gel were detected with a UV light imager and the gel pieces were subsequently stained with Coomassie to estimate the amount of LAP-A loaded on each gel piece.

### SEC-MALLS.

Purified and frozen *Tb*LAP-A, *Tc*LAP-A, and *Lm*LAP-A were thawed and diluted to concentrations of 0.1, 0.5, 1.0, and 3.0 mg ml^−1^, and additionally, *Tb*LAP-A was diluted to 5 mg ml^−1^. The LAP-As were incubated at the respective concentrations for a minimum of 3 h at room temperature to allow potential concentration-dependent oligomeric state changes. The samples were injected automatically into a SEC-MALLS system (Wyatt Dawn HELEOS-II 18-angle light scattering detector and Wyatt Optilab rEX refractive index monitor linked to a Shimadzu high-performance liquid chromatography system comprising LC-20AD pump, a SIL-20A Autosampler, and an SPD20A UV/Vis detector) with a Superdex 200 10/300 size exclusion column (GE Healthcare) and a running buffer containing 10 mM Tris (pH 8.0), 200 mM NaCl, and 1 mM DTT. The data were analyzed with the ASTRA software (version 5.3.4.14), and the molecular mass of each sample was calculated.

### Crystallization, data collection, and structure determination.

Prior to crystallization, the LAP-As were thawed at room temperature and preincubated with the desired metal salts for 2 h and subsequently with ligand for another 2 h on ice. Precipitated protein was removed by centrifugation, and the final protein concentration was determined. All crystals were grown at 20°C. Crystals were optimized by sitting- and hanging-drop vapor diffusion with a drop size of 1 μl of protein plus 1 μl of well solution. For *Lm*LAP-A, the additive screen (Hampton Research) was used to identify a compound that enhanced the diffraction of the crystals. The conditions leading to LAP-A crystals that allowed structure solution and the ligands bound in the active sites of these structures are listed in Table S1. The crystal of apo *Tb*LAP-A was cryoprotected in well solution containing 20% (vol/vol) glycerol prior to vitrification. The other LAP-A crystals were not cryoprotected.

All computations were carried out with programs of the *CCP*4 suite (44), unless stated otherwise. The data were processed with MOSFLM (45) and AIMLESS (46). Crystallographic data and statistics are shown in Tables S2 to S5.

The structures were solved by MR with PHASER (47) or MOLREP (48). The highest structural homology to *Tb*LAP-A was predicted to be the *Caenorhabditis elegans* LAP by BLAST searching against entries in the PDB, and the protomer of PDB entry 2HB6 was used in MR to generate initial phases from which the *Tb*LAP-A model was built. For all subsequent LAP-A structures, the first *Tb*LAP-A model (apo) was used for MR. Model building was carried out with BUCCANEER (49), and manual fitting of the electron density maps and model building and fitting of the ligands were carried out in Coot (50). The structures were refined with REFMAC5 (51) and validated with Coot and MolProbity (52).

### Identification of metal ions in the *Tb*LAP-A structure.

The crystal grown in the presence of ZnCl_2_ and l-leucine used protein treated with EDTA during purification to remove any metals, and no other metal except ZnCl_2_ was added prior to crystallization. XRF scans of this crystal were collected and analyzed with the PyMca software (53), but a specific edge scan for zinc gave no signal. X-ray diffraction data were collected at each side of the theoretical zinc edge at wavelengths of 0.9795 and 1.3051 Å and after data processing with Xia2 (54) and structure solution and refinement as described above. The reflection data from REFMAC5 (including the phases) were combined with the Friedel difference (DANO) and the standard deviation of F2 (SIGDANO) from the unmerged data file (from Xia2) by CAD from *CCP*4, and anomalous difference maps were generated with the FFT program in *CCP*4.

### Anti-*Tb*LAP antibody generation.

Ten milligrams of pure recombinant *Tb*LAP-A was lyophilized, resuspended in PBS, mixed with Freund’s adjuvant (1:1 ratio), and injected into a rabbit in four inoculations of ~400 µg of protein each to obtain an anti-*Tb*LAP polyclonal antibody. Antiserum was extracted before the inoculation and tested for the recognition of *Tb*LAP-A in *T. brucei* lysates and purified recombinant protein. The antibody was affinity purified by using pure recombinant *Tb*LAP-A coupled to Affi-Gel 15 Gel resin (Bio-Rad) in accordance with the manufacturer’s instructions. Antibody sensitivity and specificity were tested via Western blot analysis (Fig. S4).

### Generation of LAP-A-RNAi, LAP-A-dKO, LAP-A-OE, and LAP-A-myc lines.

A fragment of 465 bp (positions 10 to 475 of the coding region) of the *Tb*LAP-A open reading frame (ORF) sequence was amplified by PCR from genomic DNA (*T. brucei* Lister427) with forward primer 5′ GCGGATCCAAGCTTTACCCAAGGCCGAAGCAAAA 3′ and reverse primer 5′ GCGTTAACGGGCCCCGTGCTACAGCAGCAGCGAT 3′ containing the recognition sites (underlined) for BamHI-HindIII and HpaI-ApaI, respectively. The primers were designed by using the sequence found in the GeneDB database (Tb427tmp.02.4440) and validated with the TrypanoFAN-RNAit software (55). After a first cloning into the HindIII and ApaI sites of plasmid pGR19 (56), the fragment was cloned in the BamHI and HpaI restriction sites of the previous construct in the antisense orientation to create a hairpin structure of the transcribed RNA. The plasmid obtained was used to induce downregulation of *Tb*LAP-A in *T. brucei* by RNAi. To obtain stable *Tb*LAP-A depletion, two replacement cassettes for the dKOs of the gene were generated. A 538-bp fragment of the 5′ untranslated region (UTR) upstream of the *Tb*LAP-A gene (positions −61 to −599) and a 467-bp fragment of the 3′ UTR downstream of the *Tb*LAP-A gene (positions +7 to +475) were amplified from genomic DNA with 5′ UTR forward primer 5′ CGCGGCCGCGGGAGGCGGTAATGACAAGTC 3′ (NotI restriction site underlined), 5′ UTR reverse primer 5′ GCCTCGAGGCTTCAGGTCCCCTTATGCAC 3′ (Xho1 restriction site underlined), 3′ UTR forward primer 5′ GCAGGCCTGGCGTGCAAGTATCGGTTCCC 3′ (Stu1 restriction site underlined), and 3′ UTR reverse primer 5′ GGCTAGCGCGGCCGCGTAGCGAGTAAACGAGTAAACTCA 3′ (Nhe1 and Not1 sites underlined). The 5′ UTR fragment was cloned into the pHD887 vector (57) with the XhoI and NotI endonucleases. Subsequently, the 3′ UTR fragment was cloned into the resulting vector from the previous step with StuI and NheI. The resulting vector (pHD887B/UTRs) contained the blasticidin S transferase resistance gene and was used to transform parental *T. brucei* parasites for the suppression of the first allele. To remove the second *Tb*LAP-A allele from the genome, the hygromycin phosphotransferase (HYG) selectable marker was subcloned into pHD887B/UTRs. The resulting vector, pHD887H/UTRs, was used to transfect the positive single-knockouts clones. In addition, the *Tb*LAP-A gene (Tb427tmp.02.4440) ORF was amplified by PCR and cloned into vectors pGRV23b and pGRV33 (57) with ECC forward primer 5′ GCCATATGATGCCTACTTTACCCAAGGCC 3′ (Nde1 site underlined) and ECC reverse primer 5′ GCGGATCCCTACAATTTTCGGAAGTACTCGG 3′ (BamH1 site underlined). The vectors obtained were used to transfect both BF and PF *T. brucei* parental lines.

### Trypanosome growth and transfection.

The cell lines used were *T. brucei* subsp. *brucei* BF S16 (58) and PF cell line 449 (59). The BF parasites were cultured at 37°C and 5% CO_2_ in HMI-9 medium containing 10% (vol/vol) FBS. The PF trypanosomes were cultured at 28°C in SDM-79 medium supplemented with 10% (vol/vol) FBS and 7.5 μg·ml^−1^ hemin. To generate LAP-A dKO and knockdown (RNAi) parasite cells lines, as well as LAP-A and LAP-A-myc overexpression lines, parental BF and PF cells were transfected by electroporation with 10 µg of the respective targeting vectors as previously described (58). Recombinants were selected in the presence of the corresponding drug used as a selectable marker. LAP-A-dKO and LAP-A-OE lines were also grown in leucine-free minimal medium (produced in our laboratory) supplemented with 10% (vol/vol) dialyzed FBS and 7.5 µg·ml^−1^ hemin.

### Three-dimensional immunofluorescence studies.

Immunofluorescence studies were performed as previously described (60). Briefly, for each sample 10 × 10^6^ parasites were harvested by centrifugation, resuspended in 1 ml of PBS containing MitoTracker Red (Invitrogen), and incubated in the dark at culture temperature for 15 min. After one wash with 1 ml of PBS, cells were resuspended in 4% paraformaldehyde and wash solution (PBS plus 0.2% [vol/vol] Tween 20) and fixed on a poly-l-lysine-coated glass slide. The fixed cells were washed twice and permeabilized with a solution of PBS, 1% Blocking Reagent (Roche), and 1% IGEPAL (Sigma). The immunofluorescence assay was performed with anti-*Tb*LAP-A primary polyclonal antibody (affinity purified, diluted 1:500 for overexpressing cells and 1:100 for all others) or anti-c-Myc monoclonal antibody (Sigma, 1:100) and fluorescein isothiocyanate-conjugated anti-rabbit secondary antibody (Sigma, 1:40), Alexa Fluor 488-conjugated anti-rabbit antibody (Sigma, 1:100), or Alexa Fluor 488-conjugated anti-mouse antibody (Sigma, 1:40). The slides were finally stained and mounted with Vectashield-DAPI (Vector Laboratories, Inc.). Vertical stacks of 15 to 20 slices (0.2-µm steps) were captured with either an Olympus wide-field microscope and Cell R IX81software or a Leica SP5 confocal microscope. Images were deconvolved and pseudocolored with the Huygens Essential software (version 3.3; Scientific Volume Imaging) and Fiji software (version 1.5e; ImageJ) (61), respectively. Colocalization analysis (Pearson’s and Manders’ coefficients) was performed by with the JACoP plugin (62) in ImageJ. For nutritional stress studies, 25 × 10^6^ parasites were washed twice in 1× PBS and incubated for 60, 120, or 180 min in 1× PBS. After starvation treatment, immunofluorescence assays were performed as described above.

### *In vivo* studies.

Three groups of four female C57BL/6J mice (each around 8 weeks old) were used to test *Tb*LAP-A-RNAi cell line infectivity *in vivo*. One group was infected with the parental BF line, while the two remaining groups were infected with the *Tb*LAP-A-RNAi line. Each mouse was injected intraperitoneally with 1,000 monomorphic parasites from a logarithmic culture. Drinking water containing doxycycline (1 mg·ml^−1^) was given to one of the groups infected with the *Tb*LAP-A-RNAi line to induce protein knockdown, while normal water was given to the other groups. Mouse survival was monitored for 1 week. The animal research described in this report complied with Spanish (Ley 32/2007) and European Union (2010/63/UE) legislation. The protocols used were approved by the Animal Care Committee of the Instituto de Parasitología y Biomedicina “López-Neyra,” CSIC (protocol CEEA/2014/DGP/1).

### Accession number(s).

Newly reported structures have been deposited in the PDB under accession no. 5NSK, 5NSM, 5NSQ, 5NTD, and 5NTH.
