# Supplementary figures and images for: Structural Characterization of Acidic M17 Leucine Aminopeptidases from the TriTryps and Evaluation of Their Role in Nutrient Starvation in Trypanosoma brucei
Source: mSphere. 2017 Aug 16;2(4):e00226-17. doi: 10.1128/mSphere.00226-17 (PMC5557676; doi:10.1128/mSphere.00226-17)

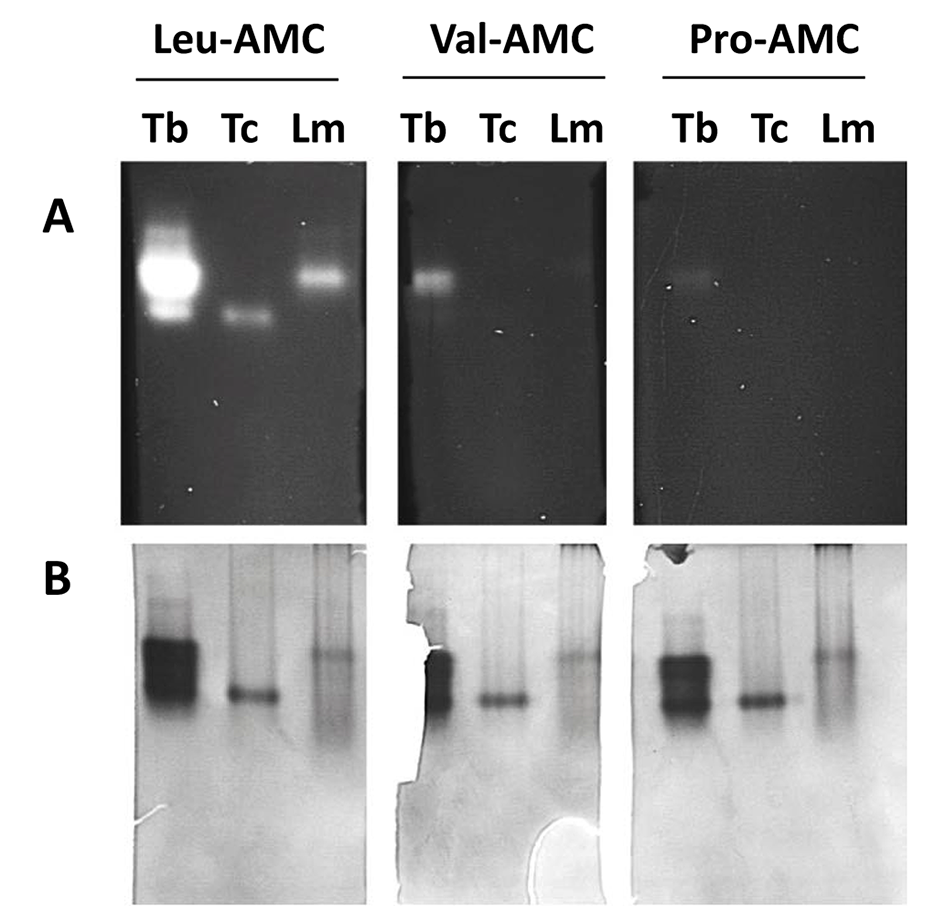

Supplement: FIG S1 [file sph004172339sf7.tif]

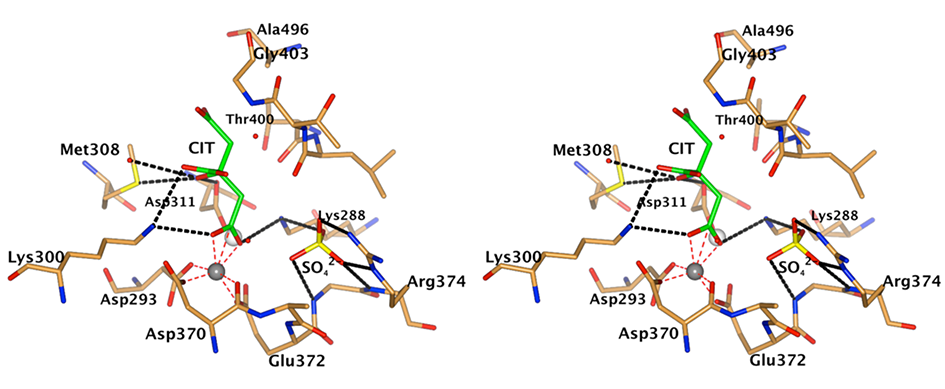

Supplement: FIG S2 [file sph004172339sf8.tif]

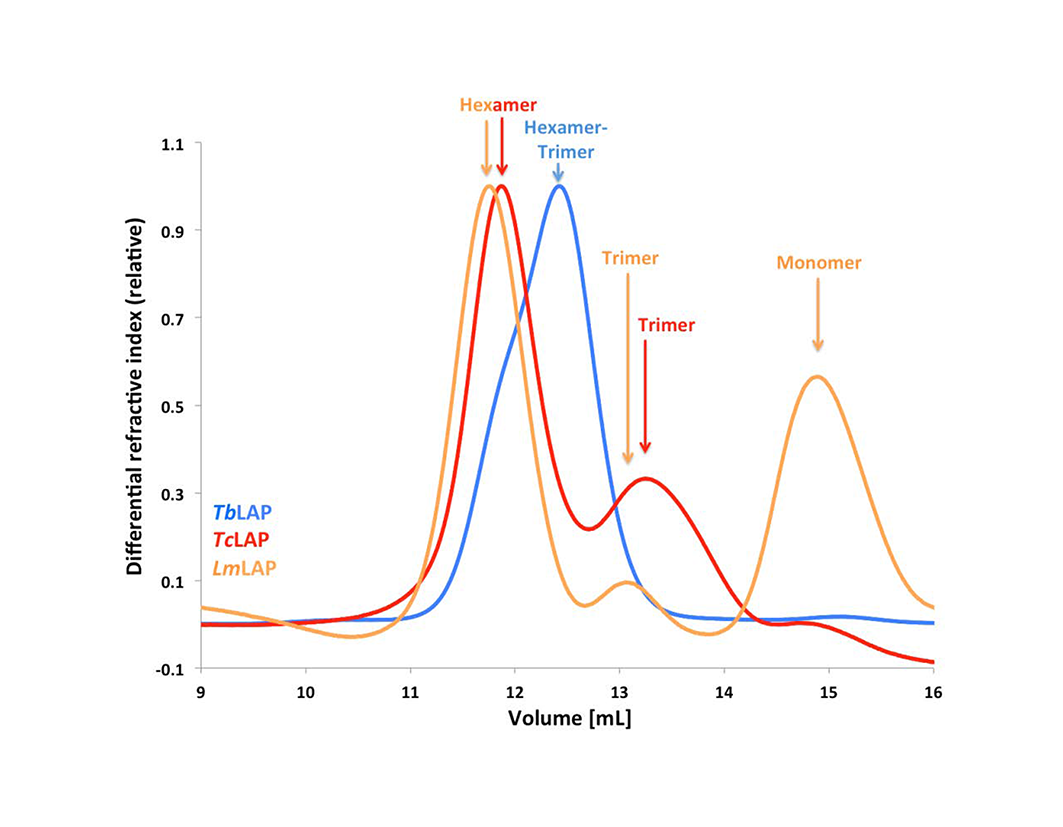

Supplement: FIG S3 [file sph004172339sf9.tif]

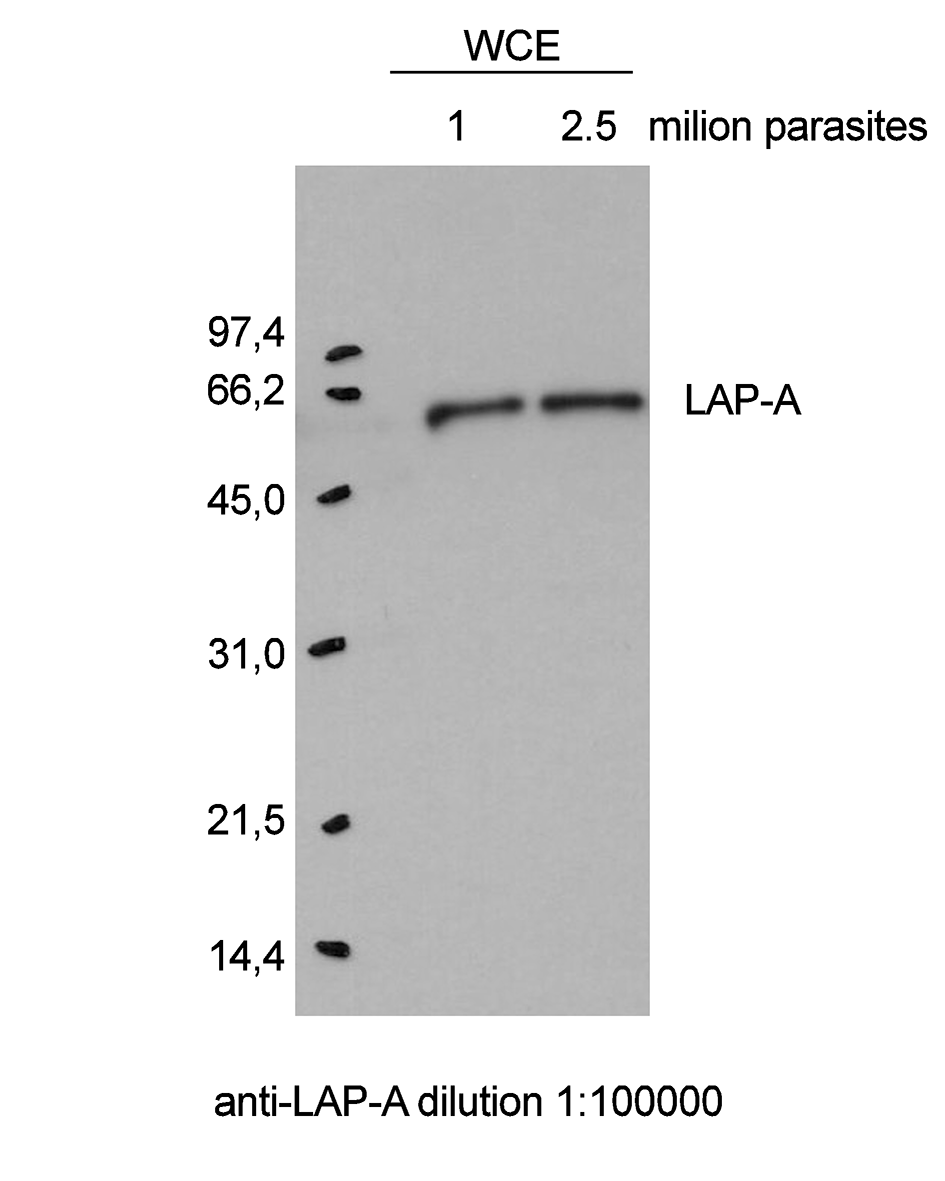

Supplement: FIG S4 [file sph004172339sf10.tif]
