# Supplementary material for: Structural Characterization of Acidic M17 Leucine Aminopeptidases from the TriTryps and Evaluation of Their Role in Nutrient Starvation in Trypanosoma brucei
Source: mSphere. 2017 Aug 16;2(4):e00226-17. doi: 10.1128/mSphere.00226-17 (PMC5557676; doi:10.1128/mSphere.00226-17)
Supplement: TABLE S1 [file sph004172339st1.docx]

Table S1: Crystallisation conditions of the LAP-As and ligands bound.

| **Protein** | **Crystal name** | **Crystallisation condition** | **Ligand bound in the active site in the structure** |
| --- | --- | --- | --- |
| *Tb*LAP-A | apo *Tb*LAP-A | 0.8 M Na citrate pH 7.0, 5 mM CoSO_4_ | - |
| *Tb*LAP-A | *Tb*LAP-A-Mn-bestatin | 0.1 M HEPES pH 7.0, 40 % (v/v) MPD, 5 mM MnCl_2_, 5 mM bestatin | Mn^2+^, HCO_3_^-^, bestatin |
| *Tb*LAP-A | *Tb*LAP-A-Mn-actinonin | 0.1 M Tris pH 8.0, 2.4 M (NH_4_)_2_SO_4_, 5 mM MnSO_4_, 5 mM actinonin | Mn^2+^, actinonin |
| *Tb*LAP-A | *Tb*LAP-A-Mn | 0.1 M Tris pH 6.8, 0.2 M MgCl_2_, 11 % (w/v) PEG 8000, 10 μM ZnCl_2_, 10 mM L-leucine | Mn^2+^, HCO_3_^-^ |
| *Tc*LAP-A | *Tc*LAP-A-Mn-citrate | 0.1 M Phosphate-citrate pH 4.2, 40 % (v/v) PEG 300, 5 mM MnSO_4_, 5 mM amastatin | Mn^2+^, SO_4_^2-^, citrate |
| *Tc*LAP-A | apo *Tc*LAP-A | 0.1 M Citric acid pH 4.0, 1.6 M (NH_4_)_2_SO_4_, 1 mM MnCl_2_, 5 mM actinonin | SO_4_^2-^ |
| *Lm*LAP-A | *Lm*LAP-A-Mn-actinonin | 0.8 M Succinic acid pH 7.0, 3 % (w/v) 1,5-Diamino-pentane di-hydrochloride, 5 mM MnSO_4_, 5 mM actinonin | Mn^2+^, HCO_3_^-^, actinonin |
