# Supplementary material for: Structural Characterization of Acidic M17 Leucine Aminopeptidases from the TriTryps and Evaluation of Their Role in Nutrient Starvation in Trypanosoma brucei
Source: mSphere. 2017 Aug 16;2(4):e00226-17. doi: 10.1128/mSphere.00226-17 (PMC5557676; doi:10.1128/mSphere.00226-17)
Supplement: TABLE S2 [file sph004172339st2.docx]

Table S2. Crystallographic data and statistics for TbLAP-A crystals.

|  | **apo TbLAP-A** | **TbLAP-A-Mn** | **TbLAP-A-Mn** |
| --- | --- | --- | --- |
| **Data collection** |  |  |  |
| Diffraction Source | DLS beamline I04 | DLS beamline I04 | DLS beamline I04 |
| Wavelength (Å) | 0.9795 | 0.9795 | 1.3052 |
| Temperature (K) | 100 | 100 | 100 |
| Detector | Pilatus 6M-F | Pilatus 6M-F | Pilatus 6M-F |
| Rotation range per image (°) | 0.5 | 0.2 | 0.2 |
| Total Rotation range (°) | 180 | 220 | 220 |
| **Crystal data** |  |  |  |
| Space group | P2_1_2_1_2_1_ | P2_1_ | P2_1_ |
| a, b, c (Å) | 162.33, 162.45, 176.99 | 87.37, 143.8, 270.1 | 87.44, 144.2, 270.4 |
| α, β, γ (°) | 90, 90, 90 | 90, 95.47, 90 | 90, 95.43, 90 |
| Resolution (Å) | 72.61-2.60 (2.64-2.60) | 32.73-2.40 (2.44-2.40) | 49.19-2.50 (2.54-2.50) |
| Total Reflections | 1065695 (53405) | 803022 (32773) | 918116 (46584) |
| Unique Reflections | 143992 (7106) | 244412 (11362) | 227280 (11336) |
| Completeness (%) | 100 (100) | 94.5 (88.7) | 98.6 (99.8) |
| Redundancy | 7.4 (7.5) | 3.3 (2.9) | 4.0 (4.1) |
| R_merge_ | 0.165 (1.019) | 0.117 (0.351) | 0.056 (0.285) |
| [I/σ (I)] | 9.5 (2.0) | 5.7 (2.0) | 14.1 (4.1) |
| Matthew’s coefficient | 3.51 | 2.40 | 2.57 |
| **Refinement statistics** |  |  |  |
| Reflections, working set | 136637 | 232199 | - |
| Reflections, test set | 7257 | 11981 | - |
| Resolution Range (Å) | 72.61-2.60 | 32.73-2.40 | - |
| R-factor | 0.1911 | 0.2059 | - |
| R_free_ | 0.2109 | 0.2250 | - |
| **No. of non-H atoms** |  |  |  |
| Protein | 22152 | 45624 | - |
| Ligands | - | 72 | - |
| Water | 500 | 661 | - |
| **Mean B factors (Å^2^)** |  |  |  |
| Protein | 29.0 | 25.7 | - |
| Ligands | - | 30.5 | - |
| Water | 31.4 | 28.9 | - |
| **RMS deviation from ideal** |  |  |  |
| Bond length (Å) | 0.0163 | 0.0140 | - |
| Bond angles (°) | 1.5325 | 1.3961 | - |
| **Ramachandran Plot (%)** |  |  |  |
| Residues in favored region | 97.88 | 97.68 | - |
| Residues in allowed region | 2.02 | 2.06 | - |
| Outliers | 0.10 | 0.26 | - |
| Molprobity score | 0.92 | 1.14 | - |
| Poor Rotamers (%) | 1.19 | 2.04 | - |
| **PDB ID** | **5NSK** | **5NSM** | **-** |
