# Supplementary material for: Structural Characterization of Acidic M17 Leucine Aminopeptidases from the TriTryps and Evaluation of Their Role in Nutrient Starvation in Trypanosoma brucei
Source: mSphere. 2017 Aug 16;2(4):e00226-17. doi: 10.1128/mSphere.00226-17 (PMC5557676; doi:10.1128/mSphere.00226-17)
Supplement: TABLE S3 [file sph004172339st3.docx]

Table S3: Crystallographic data and statistics for *Tb*LAP-A-complex crystals.

|  | ***Tb*LAP-A-Mn-bestatin** | ***Tb*LAP-A-Mn-actinonin** |
| --- | --- | --- |
| **Data collection** |  |  |
| Diffraction Source | DLS beamline I03 | DLS beamline I04-1 |
| Wavelength (Å) | 0.97625 | 0.9200 |
| Temperature (K) | 100 | 100 |
| Detector | Pilatus 6M | Pilatus 2M |
| Rotation range per image (°) | 0.2 | 0.2 |
| Total Rotation range (°) | 220 | 180 |
| **Crystal data** |  |  |
| Space group | P2_1_ | P2_1_ |
| a, b, c (Å) | 87.26, 143.45, 268.03 | 95.91, 165.73, 121.98 |
| α, β, γ (°) | 90, 95.54, 90 | 90, 112.45, 90 |
| Resolution (Å) | 80.33-2.30 (2.34-2.30) | 41.50-3.00 (3.07-3.00) |
| Total Reflections | 1132102 (52607) | 217196 (14160) |
| Unique Reflections | 290030 (14130) | 68219 (4424) |
| Completeness (%) | 99.7 (99.0) | 97.0 (98.0) |
| Redundancy | 3.9 (3.7) | 3.2 (3.2) |
| R_merge_ | 0.091 (0.523) | 0.166 (0.440) |
| [I/σ (I)] | 9.1 (2.1) | 4.5 (2.0) |
| Matthew’s coefficient | 2.51 | 2.70 |
| **Refinement statistics** |  |  |
| Reflections, working set | 275670 | 63948 |
| Reflections, test set | 14322 | 3327 |
| Resolution Range (Å) | 80.33-2.30 | 41.50-3.00 |
| R-factor | 0.1887 | 0.2646 |
| R_free_ | 0.2138 | 0.2964 |
| **No. of non-H atoms** |  |  |
| Protein | 45631 | 22512 |
| Ligands | 436 | 174 |
| Water | 2798 | 34 |
| **Mean B factors (Å^2^)** |  |  |
| Protein | 22.3 | 37.2 |
| Ligands | 47.3 | 34.0 |
| Water | 37.2 | 6.0 |
| **RMS deviation from ideal** |  |  |
| Bond length (Å) | 0.0120 | 0.0109 |
| Bond angles (°) | 1.4027 | 1.4433 |
| **Ramachandran Plot (%)** |  |  |
| Residues in favored region | 97.27 | 97.75 |
| Residues in allowed region | 2.52 | 2.06 |
| Outliers | 0.21 | 0.19 |
| Molprobity score | 1.21 | 1.15 |
| Poor Rotamers (%) | 2.37 | 1.77 |
| **PDB ID** | **5NTD** | **5NSQ** |
