# Supplementary material for: Structural Characterization of Acidic M17 Leucine Aminopeptidases from the TriTryps and Evaluation of Their Role in Nutrient Starvation in Trypanosoma brucei
Source: mSphere. 2017 Aug 16;2(4):e00226-17. doi: 10.1128/mSphere.00226-17 (PMC5557676; doi:10.1128/mSphere.00226-17)
Supplement: TABLE S4 [file sph004172339st4.docx]

Table S4: Crystallographic data and statistics for the *Tc*LAP-A crystals.

|  | ***Tc*LAP-A-Mn-citrate** | **apo *Tc*LAP-A** |
| --- | --- | --- |
| **Data collection** |  |  |
| Diffraction Source | DLS beamline I04 | DLS beamline I04 |
| Wavelength (Å) | 0.9795 | 0.9795 |
| Temperature (K) | 100 | 100 |
| Detector | Pilatus 6M-F | Pilatus 6M-F |
| Rotation range per image (°) | 0.5 | 0.2 |
| Total Rotation range (°) | 180 | 180 |
| **Crystal data** |  |  |
| Space group | H32 / R32 | H32 / R32 |
| a, b, c (Å) | 160.12, 160.12, 203.67 | 161.42, 161.42, 205.11 |
| α, β, γ (°) | 90, 90, 120 | 90, 90, 120 |
| Resolution (Å) | 57.31-2.32 (2.40-2.32) | 48.14-2.30 (2.38-2.30) |
| Total Reflections | 477971 (47316) | 380916 (39032) |
| Unique Reflections | 43546 (4249) | 43976 (4437) |
| Completeness (%) | 100 (100) | 96.2 (100) |
| Redundancy | 11.0 (11.1) | 8.7 (8.8) |
| R_merge_ | 0.188 (1.202) | 0.181 (0.987) |
| [I/σ (I)] | 11.0 (2.1) | 9.1 (2.4) |
| Matthew’s coefficient | 2.28 | 2.34 |
| **Refinement statistics** |  |  |
| Reflections, working set | 41361 | 41496 |
| Reflections, test set | 2183 | 2061 |
| Resolution Range (Å) | 57.31-2.32 | 48.14-2.30 |
| R-factor | 0.1655 | 0.2092 |
| R_free_ | 0.2100 | 0.2591 |
| **No. of non-H atoms** |  |  |
| Protein | 7648 | 7537 |
| Ligands | 51 | 20 |
| Water | 382 | 139 |
| **Mean B factors (Å^2^)** |  |  |
| Protein | 26.89 | 26.07 |
| Ligands | 47.69 | 41.65 |
| Water | 33.17 | 25.54 |
| **RMS deviation from ideal** |  |  |
| Bond length (Å) | 0.0135 | 0.0123 |
| Bond angles (°) | 1.5425 | 1.4020 |
| **Ramachandran Plot (%)** |  |  |
| Residues in favored region | 95.95 | 96.27 |
| Residues in allowed region | 3.85 | 3.54 |
| Outliers | 0.20 | 0.20 |
| Molprobity score | 1.31 | 1.21 |
| Poor Rotamers (%) | 1.86 | 0.90 |
| **PDB ID** | **5NTG** | **5NTF** |
