# Supplementary material for: Structural Characterization of Acidic M17 Leucine Aminopeptidases from the TriTryps and Evaluation of Their Role in Nutrient Starvation in Trypanosoma brucei
Source: mSphere. 2017 Aug 16;2(4):e00226-17. doi: 10.1128/mSphere.00226-17 (PMC5557676; doi:10.1128/mSphere.00226-17)
Supplement: TABLE S5 [file sph004172339st5.docx]

Table S5: Crystallographic data and statistics for the *Lm*LAP-A-Mn-actinonin.

|  | ***Lm*LAP-A-Mn-actinonin** |
| --- | --- |
| **Data collection** |  |
| Diffraction Source | DLS beamline I24 |
| Wavelength (Å) | 0.97781 |
| Temperature (K) | 100 |
| Detector | Pilatus 6M |
| Rotation range per image (°) | 0.2 |
| Total Rotation range (°) | 100 |
| **Crystal data** |  |
| Space group | P321 |
| a, b, c (Å) | 116.44, 116.44, 91.49 |
| α, β, γ (°) | 90, 90, 120 |
| Resolution (Å) | 38.11-2.50 (2.60-2.50) |
| Total Reflections | 125585 (14243) |
| Unique Reflections | 21850 (2528) |
| Completeness (%) | 87.8 (90.2) |
| Redundancy | 5.7 (5.6) |
| R_merge_ | 0.150 (0.747) |
| [I/σ (I)] | 8.2 (2.0) |
| Matthew’s coefficient | 3.09 |
| **Refinement statistics** |  |
| Reflections, working set | 20818 |
| Reflections, test set | 1031 |
| Resolution Range (Å) | 38.11-2.50 |
| R-factor | 0.1940 |
| R_free_ | 0.2449 |
| **No. of non-H atoms** |  |
| Protein | 3893 |
| Ligands | 33 |
| Water | 50 |
| **Mean B factors (Å^2^)** |  |
| Protein | 23.8 |
| Ligands | 43.1 |
| Water | 27.5 |
| **RMS deviation from ideal** |  |
| Bond length (Å) | 0.0129 |
| Bond angles (°) | 1.6417 |
| **Ramachandran Plot (%)** |  |
| Residues in favored region | 95.14 |
| Residues in allowed region | 4.67 |
| Outliers | 0.19 |
| Molprobity score | 1.76 |
| Poor Rotamers (%) | 2.26 |
| **PDB ID** | **5NTH** |
