# Supplementary material for: Structural Characterization of Acidic M17 Leucine Aminopeptidases from the TriTryps and Evaluation of Their Role in Nutrient Starvation in Trypanosoma brucei
Source: mSphere. 2017 Aug 16;2(4):e00226-17. doi: 10.1128/mSphere.00226-17 (PMC5557676; doi:10.1128/mSphere.00226-17)
Supplement: TABLE S6 [file sph004172339st6.docx]

Table S6: Expected molecular masses for different LAP-A oligomers.

|  | **Monomer [kDa]** | **Dimer [kDa]** | **Trimer [kDa]** | **Hexamer [kDa]** |
| --- | --- | --- | --- | --- |
| *Tb*LAP-A | 55.4 | 110.8 | 166.2 | 332.4 |
| *Tc*LAP-A | 55.9 | 111.8 | 167.7 | 335.4 |
| *Lm*LAP-A | 57.1 | 114.2 | 171.3 | 342.6 |
